# Snacks Fortified with Protein Concentrate from Spotted Goatfish (*Pseudupeneus maculatus*) and Passion Fruit (*Passiflora edulis*) Shell Flour

**DOI:** 10.17113/ftb.63.04.25.8776

**Published:** 2025-12-26

**Authors:** Ana Beatriz Benevides, Rodrigo Pinheiro Crasto Amaral, Eloá Dandara Carvalho da Silva, Maria Inês Sucupira Maciel, Neide Kazue Sakugawa Shinohara, Maria Beatriz de Abreu Gloria, Paulo Roberto Campagnoli de Oliveira Filho

**Affiliations:** 1 Department of Consumer Sciences, Federal Rural University of Pernambuco - UFRPE, Rua Dom Manuel de Medeiros, s/n, CEP 52171-900, Recife, PE, Brazil; 2 Department of Fisheries and Aquaculture, Federal Rural University of Pernambuco - UFRPE, Rua Dom Manuel de Medeiros, s/n, CEP 52171-900, Recife, PE, Brazil; 3 Department of Rural Technology, Federal Rural University of Pernambuco - UFRPE, Rua Dom Manuel de Medeiros, s/n, CEP 52171-900, Recife, PE, Brazil; 4 Postgraduate Program in Food Science, Faculty of Pharmacy, Federal University of Bahia – UFBA, Rua Augusto Viana, s/n - Palácio da Reitoria, CEP 40110-909, Salvador, BA, Brazil

**Keywords:** added value, fish co-products, protein supplementation, fiber supplementation, ready-to-eat foods, sustainability

## Abstract

**Research background:**

Spotted goatfish (*Pseudupeneus maculatus*) is of significant economic importance on the coast of Pernambuco, Brazil, being relevant in domestic and export markets. The fish is exported in different forms, whole, gutted, and as fillets, generating protein-rich waste. This study aims to produce a protein concentrate from spotted goatfish and add value by developing a nutritious, high-protein snack. In addition, passion fruit peel flour was used to improve the fiber content of the product.

**Experimental approach:**

The snacks were formulated with sour cassava starch, corn meal, condiments, 0 or 5 % spotted goatfish protein concentrate, and 0 or 2 % passion fruit peel flour. The physicochemical, microbiological and sensory properties of the products were compared.

**Results and conclusions:**

The products met microbiological standards for quality and safety. The snacks with added spotted goatfish protein concentrate had higher protein content than the control. Additionally, the use of passion fruit peel flour improved the texture and acceptability of the snack.

**Novelty and scientific contribution:**

The scientific contribution of this study is the improvement of snacks using co-products from the fish and juice industries, resulting in a product with improved nutritional quality in terms of protein and fiber. In addition, the use of agricultural waste supports greater sustainability.

## INTRODUCTION

Fish are sources of important nutrients, including protein and long-chain polyunsaturated fatty acids, namely eicosapentaenoic acid and docosahexaenoic acid (EPA/DHA), which are associated with reduced heart disease risk (*1*). Although fish production in Brazil has increased significantly in the last decades, fish consumption is still low at nearly 10 kg per capita/year (*2*), lower than that recommended by the World Health Organization (12 kg per capita/year) (*3*) and the world average of 20.6 kg per capita per year in 2021 (*2*). Several factors contribute to this low consumption, including cultural factors, high cost, difficulty in preparation, and poor conservation quality of fish. Expanding the use of processing technologies and offering consumers more elaborate and attractive products that are quick and easy to prepare, such as nuggets, pâtés, snacks, and other ready-to-eat products, are ways to stimulate fish consumption (*4*). Including smaller fish with low commercial value and incorporating by-products from the fish industry into newly developed products are also crucial for enhancing sustainability.

Spotted goatfish (*Pseudupeneus maculatus*) is a fishing resource of great economic importance for artisanal fisheries in the State of Pernambuco, Brazil (*5*). The commercial destinations for this species are regional open-air markets, fishmongers and the export market, mainly to the United States and Europe (*6*). This fish is exported whole, gutted, or as fillets, generating waste that could be utilized to prevent pollution and add value to the fish supply chain (*7*).

The use of by-products from fish processing has increased in recent decades to mitigate the negative environmental impacts caused by direct disposal, while providing economic benefits and expanding food production (*8*). A co-product obtained from fish processing by-products is fish protein concentrate, which has an average of 75 % protein, is chemically stable, has low moisture and fat content, is highly digestible, can be deodorized, is easy to store, and is of low cost. It is a dehydrated and minced product with a high hydration capacity, making it suitable for inclusion in food products (*9*).

Yellow passion fruit (*Passiflora edulis*) is the most widely cultivated type of passion fruit in Brazil, grown in more than 27 states, including Pernambuco. The industrial processing of passion fruit is generally focused on juice and nectar production. In this process, 54,000 tons of by-products, such as seeds and peels, are generated annually in Brazil (*10*, *11*). Passion fruit peel flour can be used as an ingredient in the preparation of functional foods, replacing conventional flour, adding technological properties to the product and ensuring the utilization of waste (*12*, *13*).

Some studies have already investigated the production of snacks with fish meat. For example, the optimum conditions for the development and study of the shelf life of extruded corn snacks with shrimp powder were evaluated (*14*). It was found that it is possible to produce snacks with the addition of shrimp powder due to the good technological results, as well as being healthier than traditional snacks and having a shelf life of up to 6 months when stored at room temperature. Another study evaluated the sensory acceptance of snacks containing between 3 and 9 % fish protein (*15*). The study showed that it is possible to add up to 7 % fish protein while maintaining good sensory characteristics in terms of smell, texture, taste and overall acceptance. The influence of adding minced fish or freeze-dried fish protein to extruded corn snacks was also evaluated in terms of physicochemical, microbial, and sensory aspects during six months of storage at room temperature (*16*). The study showed that extruding corn with minced fish or freeze-dried fish protein produces protein-rich products with a shelf life of five to six months, making them a good option for providing consumers with nutritious snacks. However, despite these studies, there are still no reports of snacks fortified with spotted goatfish protein concentrate and passion fruit peel flour. Therefore, this study aims to develop a functional snack with higher protein and fiber content using residues from the agro-industrial processing (spotted goatfish flour and passion fruit peel flour).

## MATERIALS AND METHODS

### Spotted goatfish protein concentrate

Spotted goatfish were purchased from local stores and kept frozen until processing. The fish were washed with chlorinated water to remove surface mucus, pre-processed (scaling, beheading, gutting and skinning) and filleted as described by Santos *et al.* (*17*). The meat was then mechanically separated using a mechanical deboning machine (PV 150; PV Máquinas^®^, Chapecó, Brazil) and washed using *m*(cold water):*m*(meat)=3:1. Thus obtained meat was used to produce the spotted goatfish protein concentrate type A, as described by Amaral *et al.* (*18*). The material was stirred for 2 min in a planetary dough mixer (Fleetwood BPS-12; Skymsen^®^, Brusque, Brazil) and left to rest for 3 min. The fat from the supernatant was then manually removed with a sieve, the mechanically separated meat was filtered in a nylon bag (porosity of 0.042 mm^2^), and manually pressed until the excess water was removed. The moisture was controlled by weighing the product before and after washing the meat. This same procedure was repeated once more, totaling two washing cycles. The third meat washing cycle was carried out with a 0.05 % phosphoric acid solution to deodorize the product and to reach the isoelectric point (pH close to 5.0). Another water washing cycle was performed, totaling four cycles. Then, the mechanically separated meat was placed on aluminum trays in thin layers and dried in an oven at 65 °C for 15 h. The remaining fat in the dried material was extracted with ethanol (1:2, mechanically separated meat/ethanol), and next, it was dried in an oven at 65 °C for 3 h to remove the remaining fat. Then, the material was crushed, sieved through 20- and 35-mesh sieves, packed in Ziploc^®^ bags, and stored at -20 °C until analysis.

### Passion fruit peel flour

Passion fruits (*Passiflora edulis*) were purchased from local stores. The peels were washed, and the pulp and film were removed and disinfected with chlorine (200 mg/L). Subsequently, the peels were macerated in drinking water twice for 12 h at 6 °C to eliminate bitter taste. Then, the flour was produced as described by Coelho *et al.* (*10*). After maceration, the peels were dried in a forced air circulation oven (TE-394/3; Tecnal^®^, Piracicaba, Brazil) at 70 °C for 12 h, cooled and milled in a food processor (Viva Collection RI 7761; Philips Walita^®^, São Paulo, Brazil). The flour was sieved (Bertel ISO 3310/1; Bertel Indústria Metalúrgica Ltda, Caieiras, Brazil) into particle sizes ≤425 μm.

### Formulation and production of snacks

The snacks were formulated with different mass fractions of spotted goatfish protein concentrate, passion fruit peel flour, sour cassava starch, corn meal, salt and black pepper (Table 1). The condiments were added in the same mass fractions to all formulations: 1 % salt and 0.1 % black pepper. The amount of water added was standardized at 40 % of the formulations.

**Table 1:** Formulations to produce snacks without (control) and with spotted goatfish protein concentrate and/or passion fruit peel flour

		Formulation			
Ingredient	*w*(ingredient)/%	Control	Spotted goatfish protein concentrate	Passion fruit peel flour	Spotted goatfish protein concentrate and passion fruit peel flour
		*m*/g			
Sour cassava starch	-	593.4	563.4	581.4	551.4
Corn meal	-	395.6	375.6	387.6	367.6
Spotted goatfish protein concentrate	5.0	-	50.0	-	50.0
Passion fruit peel flour	2.0	-	-	20.0	20.0
Salt	1.0	10.0	10.0	10.0	10.0
Black pepper	0.1	1.0	1.0	1.0	1.0
Total		1000.0	1000.0	1000.0	1000.0

The snacks were prepared according to Netto *et al.* (*19*), with minor modifications. The corn meal was mixed with water and cooked for 5 min at 70 °C until a firm and homogeneous dough was obtained. After cooling, the dough was mixed manually with the sour cassava starch to form a uniform mass. The remaining ingredients were then added and molded into a cylindrical shape, approx. 3 cm in diameter. The dough was cooked in boiling water (100 °C) for 10 min, cooled in ice water, dried at room temperature and placed in the refrigerator (6 °C) for uniform drying (36 h). Afterwards, the dough was sliced into 3 mm thick disks, dried in an oven at 50 °C for 4 h, cooled to room temperature (25 °C) and stored frozen (-20 °C) in its raw state. Before analysis, the snacks were fried in soybean oil at 180–200 °C for 5 min.

### Proximate composition

The proximate composition of the raw materials (spotted goatfish protein concentrate and passion fruit peel flour) and the snacks were determined by an official AOAC methodology: moisture content was analyzed by the gravimetric method in an oven at 105 °C until constant mass was achieved (*20*). Crude protein was determined by the Kjeldahl method (*N*×6.25) (*21*). Lipid content was measured by Soxhlet extraction with petroleum ether (*22*). Ash was quantified after incineration in a muffle furnace at 550 °C for 5 h (*23*). Carbohydrate content was calculated by difference, subtracting the amounts of moisture, protein, lipids and ash from 100 g. The caloric value was calculated by multiplying the amounts of protein and carbohydrate by 4 and fat by 9, respectively (*24*).

### Water activity

The water activity (*a*_w_) was determined at a temperature of 25 °C using Aqualab CX-2 equipment (Decagon Devices^®^, Pullman, WA, USA).

### CIE Lab color characteristics

The instrumental color of the snacks was determined using a portable colorimeter (CR-400; Konica Minolta^®^, Tokyo, Japan) calibrated with a white standard before each analysis. It was operated with a xenon lamp as the light source, illuminant C (Y=92.78, x=0.3139, y=0.3200), an observation angle of 2° and a measuring area 8 mm in diameter at three points. The color was expressed using the color standards of the Commission Internationale de L'Éclairage (CIE) system: L* (lightness, from lighter (+) to darker (-)), a* (red intensity (+) to green (-)), and b* (yellow intensity (+) to blue (-)).

### Expansion of the dough

The dough was expanded by immersing the snacks in soybean oil at 180–200 °C for 5 min. The snacks (*N*=3 per treatment) were marked with three lines of the same diameter using a fine-tipped pen. The length of each line was measured before and after frying, as described by Nurul *et al.* (*25*). The percentage of expansion was calculated according to the equation described by Yu *et al.* (*26*):


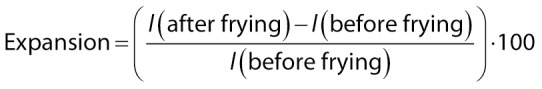
 /1/

### Microbiological analyses

The samples were collected aseptically, weighed and serially diluted in peptone saline solution for microbiological analysis. To count *Escherichia coli* and determine the presence or absence of *Salmonella*, the commercial kits Compact Dry EC^®^ and Compact Dry SL^®^ (Nissui Pharmaceutical Co. Ltd., Tokyo, Japan) were used. *Bacillus cereus* counts were determined using the APHA 31.61:2015 plating method (*27*). Molds and yeasts were counted using the APHA 21:2015 plating method (*28*). The results were compared with the requirements of the current Brazilian biscuit legislation, established by Resolution 331 (*29*) and Normative Instruction 60 (*30*).

### Sensory evaluation

The sensory evaluation was conducted with 64 untrained panelists of both genders, 36 % men and 64 % women, between 16 and 67 years old. The analysis took place in the Food Sensory Analysis Laboratory of the Department of Consumer Sciences, Federal Rural University of Pernambuco (UFRPE), Recife, Brazil, using individual cabins with white, fluorescent lighting. The snacks were fried at 180 °C for 5 min and served in white disposable cups, coded with three-digit numbers, along with water to cleanse the palate between samples. The order of presentation of the samples followed a balanced complete block design according to Wakeling *et al.* (*31*). The assessment form included the candidate profile (age and gender), the acceptance test and the preference ranking test, as described by Minim (*32*). In the acceptance test, a 9-point hedonic scale was used (1=disliked very much to 9=liked very much). The sensory attributes evaluated were appearance, color, odor, texture, flavor and overall acceptance. The ranking test was used to determine preference among the formulations and it was carried out according to Silva (*33*). The study was previously approved by the Research Ethics Committee of the Federal Rural University of Pernambuco (CAAE: 49517221.8.0000.9547).

### Statistical analysis

The experimental design was completely randomized with four formulations: (*i*) control (without spotted goatfish protein concentrate and/or passion fruit peel flour), (*ii*) addition of 5 % spotted goatfish protein concentrate and no passion fruit peel flour, (*iii*) addition of 2 % passion fruit peel flour and no spotted goatfish protein concentrate, and (*iv*) addition of 5 % spotted goatfish protein concentrate and 2 % passion fruit peel flour), with three replicates each.

The results of the laboratory analyses were initially evaluated for normality and homogeneity of variances. When these prerequisites were met, a one-way analysis of variance (ANOVA) was performed for physicochemical and sensory analyses. Subsequently, the Tukey’s multiple comparison test was applied at a 5 % significance level. Statistical analyses were carried out using the jamovi statistical program (*34*).

## RESULTS AND DISCUSSION

The *a*_w_ value and moisture and ash mass fractions of the spotted goatfish protein concentrate (Table 2) were similar to those reported by Amaral *et al.* (*18*). The protein mass fraction was higher, while the lipid mass fraction was lower than that found by Amaral *et al.* (*18*), probably due to variations in the age of the fish and the season (*35*). Spotted goatfish protein concentrate had a low mass fraction of carbohydrates (Table 2), with its caloric value increased by the amount of lipids. In their research, Correa *et al.* (*36*) prepared pirarucu (*Arapaima gigas*) flour and found a protein content of 50.5 % and a lipid content of 7.8 %. The protein content was lower than that found in this study, probably because the preparation of the flour did not include a protein concentration step. The lipid content was similar because pirarucu is a low-fat fish.

**Table 2:** Proximate composition, caloric value, water activity, and CIE L*, a*, b* color characteristics of the passion fruit peel flour and spotted goatfish protein concentrate

Parameter	Passion fruit peel flour	Spotted goatfish protein concentrate
	*w*/(g/100 g)	
Moisture	10.7±0.2	3.6±0.2
Protein	5.7±0.2	81.9±0.7
Lipid	2.26±0.08	7.9±1.2
Ash	6.1±0.2	3.0±0.1
Carbohydrate	74.6±0.3	3.4±0.5
	*E*/(kcal/100 g)	
Caloric value	341.4±0.3	413±7
*a*_w_	0.63±0.00	0.50±0.01
L*	74.0±2.1	58.6±2.1
a*	4.0±0.2	3.5±0.4
b*	28.3±0.2	29.5±0.6

The instrumental color of the spotted goatfish protein concentrate in this study differed from that obtained by Amaral *et al.* (*18*), with a higher L* value (lighter), a lower a* value (less red) and a higher b* value (more yellowish) (Table 2). As the used methodology was similar, the variations may be related to the natural color variation of this fish species.

As shown in Table 2, passion fruit peel flour had low moisture, protein, and lipid mass fraction and water activity value. These results are similar to those published by Cazarin *et al.* (*12*) and Garcia *et al.* (*37*).

The lightness of the snacks was higher in the control, while in the other formulations, the L* values were lower, with no significant difference among them (Table 3). Huda *et al.* (*38*) reported that the type of flour used in fish snacks affects the lightness of the product. Furthermore, the addition of spotted goatfish protein concentrate and the addition of spotted goatfish protein concentrate with passion fruit peel flour also decreased the L* value. According to Nurul *et al.* (*25*), the higher the fish meat content, the lower the L* value in snacks, as fish meat contains coloring pigments, which, depending on the species, will darken the product. The lower lightness observed in the fried snacks with added spotted goatfish protein concentrate and passion fruit peel flour is a result of the darkening of proteins and carbohydrates (higher values in these formulations) due to the Maillard reaction (*39*). In general, the L* values of the snacks in this study were close to those observed by Zzaman *et al.* (*39*). The redness (a* value) of the snacks was lower in the control, and higher in the other formulations, with no significant difference among them (Table 3). The highest a* value is related to the use of passion fruit peel flour and spotted goatfish protein concentrate (formulations with passion fruit peel flour and spotted goatfish protein concentrate, and passion fruit peel flour or spotted goatfish protein concentrate) (Table 2), which have pigments that give a redder color. Ribeiro *et al.* (*40*) obtained higher a* values in pasta with the addition of yellow passion fruit peel flour. The values in this study were similar to those found by Huda *et al.* (*38*) when they analyzed fish snacks from different producers in Malaysia (average of 3.84, ranging from 1.03 to 5.89) and Tamsir *et al.* (*41*), who found values from 2.13 to 4.57. The lowest yellowness value was measured in the control sample, while the highest was in the formulation containing both spotted goatfish protein concentrate and passion fruit peel flour. The spotted goatfish protein concentrate and passion fruit peel flour formulations had intermediate values, with no significant difference. The b* values found in this study were higher than those reported by Huda *et al.* (*38*), which ranged from 7.77 to 20.62 (average of 16.12). This difference can be explained by the ingredients used in the snacks, such as corn flour, passion fruit peel flour, and spotted goatfish meat, which have high yellowness values. The greater yellowness (higher b* value) observed in fried snacks with added spotted goatfish protein concentrate and passion fruit peel flour is the result of the darkening of proteins and carbohydrates (higher values in these formulations) due to the Maillard reaction (*42*).

**Table 3:** CIE Lab color characteristics (L*, a* and b*) before and after frying and expansion of fried snacks made without (control) and with spotted goatfish protein concentrate and/or passion fruit peel flour

	Formulation			
Parameter	Control	Spotted goatfish protein concentrate	Passion fruit peel flour	Spotted goatfish protein concentrate and passion fruit peel flour
Before frying				
L*	(63.2±5.3)^a^	(65.0±2.0)^a^	(64.4±3.5)^a^	(58.2±1.8)^a^
a*	(-0.9±1.1)^b^	(2.5±0.3)^a^	(3.2±0.6)^a^	(4.0±0.3)^a^
b*	(34.3±4.9)^a^	(33.5±1.7)^a^	(33.6±3.7)^a^	(30.5±0.8)^a^
After frying				
L*	(72.5±2.5)^a^	(64.8±1.2)^b^	(63.1±1.4)^b^	(59.6±2.7)^b^
a*	(-1.9±0.4)^b^	(2.4±1.5)^a^	(3.2±0.6)^a^	(4.4±0.5)^a^
b*	(26.6±1.6)^b^	(32.2±2.2)^ab^	(32.0±2.6)^ab^	(33.6±2.1)^a^
Expansion/%	(13.4±3.4)^b^	(3.2±0.3)^c^	(23.4±8.0)^a^	(3.5±1.0)^c^

Results are expressed as mean value±standard deviation, *N*=3. Different letters in superscript in the same row indicate a significant difference according to the Tukey’s test (p<0.05)

A significant variation (p<0.05) was observed in the expansion of snacks among formulations (Table 3). Snacks made only with added passion fruit peel flour showed the greatest expansion, followed by the control sample. Passion fruit peel flour increased the linear expansion of snacks, probably due to its high carbohydrate content (Table 2). Furthermore, the greatest expansion was achieved when the starch granules in the snacks were fully expanded (*38*). Formulations containing spotted goatfish protein concentrate, as well as those with spotted goatfish protein concentrate and passion fruit peel flour, expanded less, with no significant difference (p>0.05) between these formulations (Table 3). The addition of spotted goatfish protein concentrate negatively affected product expansion. This may have resulted from the interaction of the protein with the starch granules, making starch gelatinization difficult (*39*) and thus reducing the expansion of the snacks.

However, the values found for the expansion of the snacks were low, as, according to Huda *et al.* (*38*), the expansion of fish snacks must be greater than 77 % to achieve the desirable crunchiness of the product. It is possible that during the dehydration process, there was a change in the consistency of the dough and the formation of the gel, which prevented the formation of fine muscle bundles evenly distributed in the starch gel typical of fresh fish protein (*38*).

Table 4 shows the proximate composition of the fried snacks. There was no significant difference (p>0.05) in moisture among formulations. All of them were similar to values reported in the literature (*19*, *39*).

**Table 4:** Proximate composition, caloric value and water activity of fried snacks made without (control) and with spotted goatfish protein concentrate and/or passion fruit peel flour

	Formulation			
Parameter	Control	Spotted goatfish protein concentrate	Passion fruit peel flour	Spotted goatfish protein concentrate and passion fruit peel flour
	*w*/(g/100 g)			
Moisture	(7.3±0.8)^a^	(6.5±0.2)^a^	(6.1±0.4)^a^	(6.7±0.2)^a^
Ash	(0.8±0.1)^ab^	(0.93±0.06)^a^	(0.67±0.09)^b^	(1.01±0.07)^a^
Protein	(2.2±0.4)^b^	(5.5±0.7)^a^	(2.3±0.5)^b^	(5.7±0.2)^a^
Lipid	(16.1±2.2)^a^	(11.8±1.8)^a^	(13.50±2.00)^a^	(7.1±1.4)^b^
Carbohydrate	(73.5±2.9)^a^	(75.2±2.5)^a^	(77.4±2.0)^a^	(79.4±1.7)^a^
	*E*/(kcal/100 g)			
Caloric value	(448±10)^a^	(429±9)^a^	(440±9)^a^	(404±7)^b^
*a*_w_	(0.58±0.01)^a^	(0.58±0.01)^a^	(0.56±0.01)^a^	(0.56±0.00)^a^

Results are expressed as mean value±standard deviation, *N*=3. Different letters in superscript in the same row indicate a significant difference according to the Tukey’s test (p<0.05)

Higher values of ash mass fractions were obtained in the snacks containing spotted goatfish protein concentrate alone or in combination with passion fruit peel flour than in the control sample. Baskar *et al.* (*43*) found a significant increase in the ash content in extruded snacks enriched with fish flour. A similar result was observed in a study on the fortification of biscuits with carp or shark protein concentrate (*44*). However, the ash mass fraction of the spotted goatfish protein concentrate was about half that of the passion fruit peel flour. The main difference was that the mass fraction of spotted goatfish protein concentrate in the snacks was 2.5 times higher than that of passion fruit peel flour (Table 1). Furthermore, the process of obtaining mechanically separated meat can grind bones together, increasing the amount of ash in the product (*7*). The ash values found are lower than those reported in the literature, which range from 2.3 to 8.9 % in fried fish snacks (*19*, *39*, *45*). This difference can be explained by the ingredients used, mainly the amount of salt. In the formulations mentioned above, the salt mass fraction varied between 2 and 3 %, while in this study, the salt mass fraction was 1 %. In the present study, neither monosodium glutamate nor sodium bicarbonate was used, as in the study by Zzaman *et al.* (*39*), nor was a larger number of seasonings used, as in the study by Zim *et al.* (*45*).

The highest mass fraction of protein in the snacks was found in the formulation containing spotted goatfish protein concentrate and passion fruit peel flour, followed by the formulation containing only spotted goatfish protein concentrate (Table 4). This shows the importance of adding spotted goatfish protein concentrate for protein fortification in snacks. This result supports the findings of Baskar *et al.* (*43*) and Correa *et al.* (*36*), who found an increase in the amount of protein in snacks enriched with fish flour. The protein content in fish snacks is related to the fish species and the proportion of fish meat and added starch (*42*).

The highest mass fraction of lipids was found in the control, spotted goatfish protein concentrate, and passion fruit peel flour formulations, with no significant differences among them. The lowest lipid value was found in the formulation containing spotted goatfish protein concentrate and passion fruit peel flour. The addition of fish protein concentrate reduces oil absorption during frying, which is explained by the lower linear expansion during frying (Table 3) and the resulting reduced formation of air pockets. The lipid values of fried snacks made with mechanically separated tilapia meat found by Netto *et al.* (*19*) were on average 16.53 % and were similar to those observed in the control and passion fruit peel flour formulations.

There was no significant difference among formulations regarding carbohydrates. The values found are similar to those published by Netto *et al.* (*19*), ranging from 66.74 to 75.87 %, and Huda *et al.* (*38*), which ranged from 53.62 to 80.43 %.

Among the snacks, the formulation containing spotted goatfish protein concentrate and passion fruit peel flour had a lower caloric value than the other formulations, which is explained by the lower mass fraction of lipids due to reduced oil absorption capacity caused by the lower expansion in this formulation (Table 3). The values found were similar to those reported by Netto *et al.* (*19*) in fried snacks, ranging from 446.60 to 449.50 kcal/100 g, and lower than that published by Neiva *et al.* (*46*), who found a value of 518.07 kcal/100 g.

There was no significant difference among formulations regarding water activity (Table 4). The water activity values of the snacks in this study were higher than those reported by Netto *et al.* (*19*), which were between 0.39 and 0.48, and Neiva *et al.* (*46*), who observed a water activity of 0.36 in fish snacks. Water activity values below 0.6 inhibit microbial growth (*47*) and, consequently, snacks from all formulations showed good preservation characteristics and stability. Several factors, such as the presence of starch in the formulation and the drying and frying processes, can result in low water activity in snacks (*16*).

*Salmonella* was not detected in any of the formulations. The counts of *B. cereus* and *E. coli*/g were below 2 log CFU/g, which complies with legislation (*27*, *28*). The mold and yeast counts were 1 log CFU/g in all formulations. The results were compared with established microbiological standards in Brazil, and all met the requirements for biscuits, thus proving to be safe for consumption.

**Fig. 1:**
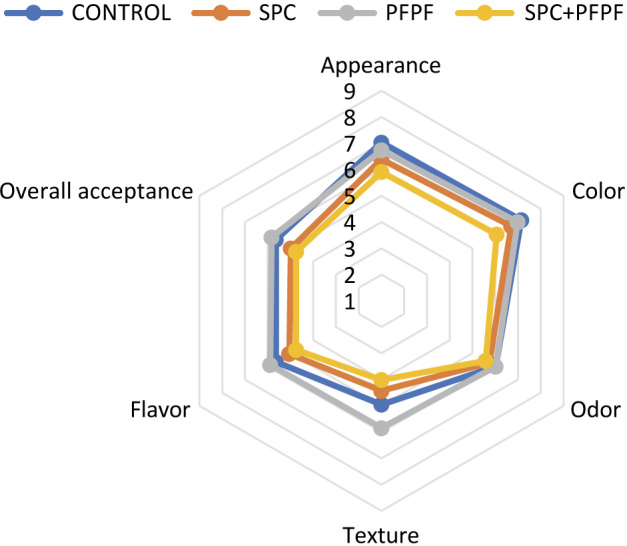
Radar graph of the sensory evaluation of snacks made without (control) and with spotted goatfish protein concentrate (SPC) and/or passion fruit peel flour (PFPF)

The appearance and color of snacks from the control, the formulation containing passion fruit peel flour, and that containing spotted goatfish protein concentrate were better accepted (Fig. 1). This shows that the appearance and color of snacks with added passion fruit peel flour and spotted goatfish protein concentrate improved when used separately. This result is shown in Table 3, where snacks containing spotted goatfish protein concentrate and passion fruit peel flour had lower lightness (L* value) and higher redness (a* value), making them darker than the other formulations. Netto *et al.* (*19*) also observed lower color scores for fish snacks with a darker color. The values reported by previous authors were similar to those obtained in the present study, between 6.0 and 7.0 (“I liked it slightly”). Zim *et al.* (*45*) observed higher scores for the orange-colored snack, with a rating of “I liked it moderately” (6.0 points), while other snacks received a “slightly liked” (5.0 points) rating. Tamsir *et al.* (*41*) also found values between 6.0 and 7.0 (“I liked it slightly”) for the appearance and color attributes of keropok-type snacks.

The odor of the snacks did not differ significantly (p>0.05) among formulations, with average scores equivalent to “I liked it slightly”. This may be because these ingredients have a mild odor. Netto *et al.* (*19*) found similar values for the odor attribute for snacks made with mechanically separated tilapia meat, ranging from 6.53 to 6.80 (“I liked it slightly”). Zim *et al.* (*45*) also obtained “I liked it slightly” ratings for fish snacks made with a traditional formula, while snacks with added seasonings were evaluated as “I liked it moderately”. Shaviklo *et al*. (*16*) studied the inclusion of minced fish or freeze-dried fish protein in snacks and did not observe variation in the odor acceptance of corn snacks.

The best texture score was given to snacks made with passion fruit peel flour. In contrast, the lowest scores were given to snacks containing spotted goatfish protein concentrate and those containing both spotted goatfish protein concentrate and passion fruit peel flour (Fig. 1). This suggests that the addition of spotted goatfish protein concentrate may have negatively affected the texture of the snacks. This result is directly related to the expansion (Table 3), where the passion fruit peel flour formulation showed the greatest expansion, and the spotted goatfish protein concentrate and spotted goatfish protein concentrate with passion fruit peel flour formulation showed the lowest expansion. Netto *et al.* (*19*) reported a decrease in the sensory acceptance of the texture attribute with an increase in the percentage of mechanically separated tilapia meat in snacks. The values they found ranged from 5.6 (40 % addition) to 7.6 (20 % addition). Tamsir *et al.* (*41*) found higher texture scores for keropok-type snacks fried in oil, between 5.0 and 6.0 (“neither like nor dislike”).

The best flavor result was for the formulation with added passion fruit peel flour, while the lowest rating was for the spotted goatfish protein concentrate with passion fruit peel flour (Fig. 1). This may indicate that the addition of spotted goatfish protein concentrate worsened the taste of the snacks, possibly because the fish flavor is not common in Brazilian snacks, leading to lower acceptance. Netto *et al.* (*19*) observed a decrease in the sensory acceptance of the flavor attribute with an increase in mechanically separated tilapia meat in snacks (inclusion from 20 to 40 %). Zim *et al.* (*45*) obtained “I liked it slightly” (5 points) evaluations for fish snacks made with a traditional formula. In comparison, snacks with added seasonings were evaluated as “I liked it moderately” (6 points). Tamsir *et al.* (*41*) found values between 6.0 and 7.0 (“I liked it slightly”) for the flavor attribute for keropok-type snacks fried in oil.

The overall acceptance of snacks was higher in the control and in the one with added passion fruit peel flour. However, the combination of spotted goatfish protein concentrate and passion fruit peel flour or only spotted goatfish protein concentrate were the least accepted (Fig. 1). Netto *et al.* (*19*) reported a decrease in overall acceptance with the inclusion of 20 to 40 % of mechanically separated tilapia meat in snacks, with values ranging from 5.9 (40 % addition) to 7.0 (20 % addition). Zim *et al.* (*45*) obtained “I liked it slightly” (5 points) ratings for fish snacks made with a traditional formula. In comparison, snacks with added seasonings were rated as “I liked it moderately” (6 points). Tamsir *et al.* (*41*) found values between 6.0 and 7.0 (“I liked it slightly”) for the overall acceptance of keropok-type snacks fried in oil. However, comparing sensory analysis results from tasters in different countries is very difficult due to the subjectivity of this type of analysis. In addition to cultural differences among panelists, factors such as seasoning and salt content affect panelists' acceptance.

The control and passion fruit peel flour formulations were preferred in the ordering (preference) test (Table 5). This result could be improved by reducing the amount of spotted goatfish protein concentrate and passion fruit peel flour in the formulation, and by adding seasonings to enhance acceptability.

**Table 5:** Ordering test (preference) of snacks made without (control) or with spotted goatfish protein concentrate and/or passion fruit peel flour

Formulation	Preference
Control	(175.0±1.1)^a^
Spotted goatfish protein concentrate	(143.0±1.1)^b^
Passion fruit peel flour	(188.0±1.0)^a^
Spotted goatfish protein concentrate and passion fruit peel flour	(124.0±1.0)^c^

Different letters in superscript indicate significant differences (Tukey’s test, p<0.05)

## CONCLUSIONS

Fortification with spotted goatfish protein concentrate improved the nutritional quality and increased the protein content of the snacks. However, these snacks showed reduced expansion and received lower texture acceptance ratings. The addition of passion fruit peel flour offered technological benefits by improving snack expansion. The developed snacks were moderately accepted by consumers. The scientific contribution of this study lies in the improvement of snacks using co-products from the fish and juice industries, resulting in a product with improved nutritional quality regarding protein and fiber. In addition, the use of agricultural waste contributes to greater sustainability.

## References

[1] Wan Y Zheng J Wang F Li D. Fish, long chain omega-3 polyunsaturated fatty acids consumption, and risk of all-cause mortality: A systematic review and dose-response meta-analysis from 23 independent prospective cohort studies. Asia Pac J Clin Nutr. 2017;26(5):939–56. 10.6133/apjcn.072017.01 28802305

[2] The state of world fisheries and aquaculture 2024. Blue Transformation in action. Rome, Italy: Food and Agriculture Organization of the United Nations (FAO); 2024. 10.4060/cd0683en

[3] Oliveira LP Souza ALM. Consumo de pescado no Brasil e ocorrências de falsificações na cadeia produtiva: Revisão (Fish consumption in Brazil and occurrences of counterfeiting in the production chain: A review). PUBVET. 2024;18(04):e1571 (in Portuguese). 10.31533/pubvet.v18n04e1571

[4] Macedo IME Andrade HA Sakugawa Shinohara NK Maciel MIS Glória MBA Oliveira Filho PRC. Influence of ultrasound on the microbiological and physicochemical stability of saramunete (*Pseudupeneus maculatus*) sausages. J Food Process Preserv. 2021;45:e15580. 10.1111/jfpp.15580

[5] Marques S, Ferreira BP. Composição e características da pesca de armadilhas no litoral Norte de Pernambuco – Brazil (Composition and characteristics of trap fishing on the northern coast of Pernambuco - Brazil). Bol Tec Cient CEPENE. 2010;18(1):49-60. Available from: https://bdc.icmbio.gov.br/items/7e7f1c4f-fa92-41c4-b11b-26b5c703c206 (in Portuguese).

[6] Cardoso L Lacerda ACF Gonçalves ELT Cadorin DI Bonfim CNC Oliveira RLM Gill metazoan parasites of the spotted goatfish *Pseudupeneus maculatus* (Ostheichthyes: Mullidae) from the Coast of Pernambuco, northeastern Brazil. Braz J Biol. 2018;78(3):414–20. 10.1590/1519-6984.166631 29160360

[7] Sá Júnior PLS Silva LJ Andrade HA Oliveira Filho PRC. Rendimento e composição centesimal de filés e carne mecanicamente separada de saramunetes (*Pseudupeneus maculatus* BLOCH, 1793) (Yield and centesimal composition of fillets and mechanically separate meat of spotted goatfish (*Pseudupeneus maculatus* Bloch, 1793)). Arq Ciên Mar. 2020;53(1):52–62 (in Portuguese). 10.32360/acmar.v53i1.42985

[8] Olsen RL Toppe J Karunasagar I. Challenges and realistic opportunities in the use of by-products from processing of fish and shellfish. Trends Food Sci Technol. 2014;36(2):144–51. 10.1016/j.tifs.2014.01.007

[9] Vidal JMA Rodrigues MCP Zapata JFF Vieira JMM. Concentrado protéico de resíduos da filetagem de tilápia-do-nilo (*Oreochromis niloticus*): Caracterização físico-química e aceitação sensorial (Protein concentrate from the residues left after filleting Nile tilapia (*Oreochromis niloticus*): Physical-chemical characterization and sensory acceptance). Rev Ciênc Agron. 2011;42(1):92–9 (in Portuguese). 10.1590/S1806-66902011000100012

[10] Coelho EM Gomes RG Machado BAS Oliveira RS Lima MS Azêvedo LC Passion fruit peel flour - Technological properties and application in food products. Food Hydrocoll. 2017;62:158–64. 10.1016/j.foodhyd.2016.07.027

[11] Córdova KRV Gama TMMTB Winter CMG Kaskantzis Neto G Freitas RJS. Características físico-químicas da casca do maracujá amarelo (*Passiflora edulis* Flavicarpa Degener) obtida por secagem (Physico-chemical characteristics of yellow passion fruit (*Passiflora edulis* Flavicarpa Degener) peel after a drying process). Bol Cent Pesqui Proc Aliment. 2005;23(2):221–30 (in Portuguese). 10.5380/cep.v23i2.4491

[12] Cazarin CBB Silva JK Colomeu TC Zollner RL Maróstica MR Junior. Capacidade antioxidante e composição química da casca de maracujá (*Passiflora edulis*) (Antioxidant capacity and chemical composition of passion fruit peel (*Passiflora edulis*)). Ciênc Rural. 2014;44(9):1699–1704 (in Portuguese). 10.1590/0103-8478cr20131437

[13] Weng M Li Y Wu L Zheng H Lai P Tang B Effects of passion fruit peel flour as a dietary fibre resource on biscuit quality. Food Sci Technol (Campinas). 2021;41(1):65–73. 10.1590/fst.33419

[14] Shaviklo AR Azaribeh M Moradi Y Zangeneh P. Formula optimization and storage stability of extruded puffed corn-shrimp snacks. LWT – Food Sci Technol. 2015;63(1):307–14. 10.1016/j.lwt.2015.03.093

[15] Shaviklo GR Olafsdottir A Sveinsdottir K Thorkelsson G Rafipour F. Quality characteristics and consumer acceptance of a high fish protein puffed corn-fish snack. J Food Sci Technol. 2011;48(6):668–76. 10.1007/s13197-010-0191-1 23572803 PMC3551053

[16] Shaviklo GR Thorkelsson G Rafipourd F Sigurgisladottir S. Quality and storage stability of extruded puffed corn-fish snacks during 6-month storage at ambient temperature. J Sci Food Agric. 2011;91(5):886–93. 10.1002/jsfa.4261 21384356

[17] Santos FK Vasconcelos Filho MB Vieira PHS Malheiros LS Oliveira Filho PRC. Rendimento corporal do saramunete, *Pseudupeneus maculatus* (Bloch, 1793) submetido a diferentes métodos de filetagem (Body yield of spotted goatfish *Pseudupeneus maculatus* (Bloch, 1793) subjected to different filleting methods). Arq Ciên Mar. 2016;49(2):15–22 (in Portuguese). 10.32360/acmar.v49i2.6588

[18] Amaral RPC Silva EDC Oliveira Filho PRC. Obtenção e caracterização físico-química e nutricional de concentrado proteico de resíduos de filetagem de saramunete, *Pseudupeneus maculatus* (Bloch, 1793) (Obtaining and physicochemical and nutritional characterization of protein concentrate from fillet residues of saramunete, *Pseudupeneus maculatus* (Bloch, 1793)). Arq Ciên Mar. 2021;54(2):69–80 (in Portuguese). 10.32360/acmar.v54i2.62788

[19] Cortez Netto JP Oliveira Filho PRC Lapa-Guimarães J Viegas EMM. Physicochemical and sensory characteristics of snack made with minced Nile tilapia. Food Sci Technol (Campinas). 2014;34(3):591–6. 10.1590/1678-457x.6395

[20] Official Method AOAC. 926.12. Moisture and volatile matter in oils and fats. Vacuum oven method. Rockville, MD, USA: AOAC International; 1996.

[21] Official Method AOAC. 991.20. Nitrogen (total) in milk: Kjeldahl methods. Rockville, MD, USA: AOAC International; 1994.

[22] Official Method AOAC. 920.39. Fat (crude) or ether extract in animal feed. Rockville, MD, USA: AOAC International; 1995.

[23] Official Method AOAC. 900.02. Ash of sugars and syrups. Rockville, MD, USA: AOAC International; 1996.

[24] Zenebon O, Pascuet NS, Tiglea P, editors. Physicochemical methods for food analysis. São Paulo, SP, Brazil: Instituto Adolfo Lutz; 2008 (in Portuguese).

[25] Nurul H Boni I Noryati I. The effect of different ratios of Dory fish to tapioca flour on the linear expansion, oil absorption, colour and hardness of fish crackers. Int Food Res J. 2009;16(2):159–65.

[26] Yu SY Mitchell JR Abdullah A. Production and acceptability testing of fish crackers (‘keropok’) prepared by the extrusion method. Int J Food Sci Technol. 1981;16(1):51–8. 10.1111/j.1365-2621.1981.tb00995.x

[27] Bennett RW, Tallent SM, Hait JM. *Bacillus cereus* and *Bacillus cereus* toxins. In: Salfinger Y, Tortorello ML, editors. Compendium of methods for the microbiological examination of foods. Washington, DC, USA: American Public Health Association (APHA); 2013. 10.2105/MBEF.0222.036

[28] Ryu D, Wolf-Hall C. Yeasts and molds. In: Salfinger Y, Tortorello ML, editors. Compendium of methods for the microbiological examination of foods. Washington, DC, USA: American Public Health Association (APHA); 2013. 10.2105/MBEF.0222.026

[29] Resolution of the Collegiate Board nº 331, of 23 December 2019. Provides for food microbiological standards and their application. Official Gazette of the Union. 2019;249(1):96. Available from: https://www.in.gov.br/web/dou/-/resolucao-rdc-n-331-de-23-de-dezembro-de-2019-235332272 (in Portuguese).

[30] Normative instruction of the Collegiate Board n° 60. Establishes the lists of microbiological standards for food. Offcial Gazette of the Union. 2019:249(1):133. Available from: https://bvsms.saude.gov.br/bvs/saudelegis/anvisa/2019/IN_60_2019_COMP.pdf (in Portuguese).

[31] Wakeling IN MacFie HJH. Designing consumer trials balanced for first and higher orders of carry-over effect when only a subset of *k* samples from *t* may be tested. Food Qual Prefer. 1995;6(4):299–308. 10.1016/0950-3293(95)00032-1

[32] Minim VPR, editor. Análise sensorial: estudos com consumidores (Sensory analysis. Studies with consumers). Viçosa, MG, Brazil: Editora UFV; 2018 (in Portuguese).

[33] Silva MAAP, editor. Métodos de avaliação sensorial de alimentos (Food sensory evaluation methods). Campinas, SP, Brazil: Escola de Extensão de UNICAMP; 1997 (in Portuguese).

[34] The jamovi project, v. 2.2, Sydney, Australia: jamovi; 2021. Available from: https://www.jamovi.org.

[35] Boran G Karaçam H. Seasonal changes in proximate composition of some fish species from the Black Sea. Turk J Fish Aquat Sci. 2011;11:1–5. 10.4194/trjfas.2011.0101

[36] Correa SS Oliveira GG Santos FV Coradini MF Souza Alves LF Matiucci MA Flavored Amazonic pirarucu (*Arapaima giga*) waste flour (salted and sweet) for inclusion in food products. J Food Sci Technol. 2022;59(8):3053–62. 10.1007/s13197-022-05480-7 35872727 PMC9304487

[37] Garcia MV Milani MS Ries EF. Production optimization of passion fruit peel flour and its incorporation into dietary food. Food Sci Technol Int. 2020;26(2):132–9. 10.1177/1082013219870011 31537114

[38] Huda N Leng AL Yee CX Herpandi H. Chemical composition, colour and linear expansion properties of Malaysian commercial fish cracker (keropok). Asian J Food Agro-Ind. 2010;3(5):473–82.

[39] Zzaman W Yusoff MM Yang TA. Preparation and properties of fish cracker from different freshwater fish species. Int Food Res J. 2017;24(5):1858–62.

[40] Ribeiro THS Bolanho BC Montanuci FD Ruiz SP. Physicochemical and sensory characterization of gluten-free fresh pasta with addition of passion fruit peel flour. Cienc Rural. 2018;48(12):e20180508. 10.1590/0103-8478cr20180508

[41] Tamsir MM Ramli NS Nor-Khaizura MAR Shukri R Ismail-Fitry MR. Comparison of boiling, steaming, air frying, deep-frying, microwaving and oven-cooking on quality characteristics of keropok lekor (Malaysian fish sausage). Malays Appl Biol. 2021;50(3):77–85. 10.55230/mabjournal.v50i3.2000

[42] Cheow CS Yu SY Howell NK Man YC Muhammad K. Effect of fish, starch and salt contents on the microstructure and expansion of fish crackers (‘keropok’). J Sci Food Agric. 1999;79(6):879–85. 10.1002/(SICI)1097-0010(19990501)79:6<879::AID-JSFA295>3.0.CO;2-P

[43] Baskar D Dhanapal K Madhavan N Madhavi K Kumar GP Manikandan V Proximate composition and sensory evaluation of extruded snacks enriched with fish flour and shrimp head exudate during storage conditions. J Food Process Preserv. 2022;46:e16589. 10.1111/jfpp.16589

[44] Mohamed GF Sulieman AM Soliman NG Bassiuny SS. Fortification of biscuits with fish protein concentrate. World J Dairy Food Sci. 2014;9(2):242–9.

[45] Zim AFMIU Akter A Ali MS Anik WA Ahmed S Zamri AIB. Proximate composition, texture analysis and sensory evaluation of keropok lekor formulated with herbs and spices. Food Res. 2019;3(6):635–9. 10.26656/fr.2017.3(6).050

[46] Neiva CRP Machado TM Tomita RY Furlan EF Lemos Neto MJ Bastos DHM. Fish crackers development from minced fish and starch: An innovative approach to a traditional product. Food Sci Technol (Campinas). 2011;31(4):973–9. 10.1590/S0101-20612011000400024

[47] Jatobá RF Oliveira Filho PRC. Silagem biológica elaborada com resíduos de filetagem de saramunete (*Pseudupeneus maculatus*) (Biological silage elaborated with saramunete (*Pseudupeneus maculatus*) filleting waste). Rev Bras Eng Pesca. 2017;10(1):58–68 (in Portuguese). 10.18817/repesca.v10i1.1170

